# Taurine improves the spatial learning and memory ability impaired by sub-chronic manganese exposure

**DOI:** 10.1186/1423-0127-21-51

**Published:** 2014-05-24

**Authors:** Cai-Ling Lu, Shen Tang, Zhi-Juan Meng, Yi-Yuan He, Ling-Yong Song, Yin-Pin Liu, Ning Ma, Xi-Yi Li, Song-Chao Guo

**Affiliations:** 1 Department of Food and Nutrition, School of Public Health, Guangxi Medical University, 22 Shuangyong Road, 530021 Nanning, Guangxi, P.R. China; 2 Department of Immunology, School of Preclinical medicine, Guangxi Medical University, 22 Shuangyong Road, 530021 Nanning, Guangxi, P.R. China; 3 China Tobacco GuangXi Industrial CO. LTD, Nanning, China; 4 Mie University, Faculty of Medicine, Suzuka, Mie 510-0293, Japan

## Abstract

****Background**:**

Excessive manganese exposure induced cognitive deficit. Several lines of evidence have demonstrated that taurine improves cognitive impairment induced by numerous neurotoxins. However, the role of taurine on manganese-induced damages in learning and memory is still elusive. This goal of this study was to investigate the beneficial effect of taurine on learning and memory capacity impairment by manganese exposure in an animal model.

**Results:**

The escape latency in the Morris Water Maze test was significantly longer in the rats injected with manganese than that in the rats received both taurine and manganese. Similarly, the probe trial showed that the annulus crossings were significantly greater in the taurine plus manganese treated rats than those in the manganese-treated rats. However, the blood level of manganese was not altered by the taurine treatment. Interestingly, the exposure of manganese led to a significant increase in the acetylcholinesterase activity and an evidently decrease in the choline acetyltransferase activity, which were partially restored by the addition of taurine. Additionally, we identified 9 differentially expressed proteins between the rat hippocampus treated by manganese and the control or the manganese plus taurine in the proteomic analysis using the 2-dimensional gel electrophoresis followed by the tandem mass spectrometry (MS/MS). Most of these proteins play a role in energy metabolism, oxidative stress, inflammation, and neuron synapse.

**Conclusions:**

In summary, taurine restores the activity of AChE and ChAT, which are critical for the regulation of acetylcholine. We have identified seven differentially expressed proteins specifically induced by manganese and two proteins induced by taurine from the rat hippocampus. Our results support that taurine improves the impaired learning and memory ability caused by excessive exposure of manganese.

## Background

Manganese (Mn) is an essential nutrient involved in the regulation of multiple metabolic pathways. However, Mn can be toxic to many organs at a high concentration. The brain is particularly susceptible to the Mn toxicity. Excessive occupational or environmental Mn exposure is associated with multiple neurotoxic effects. Early neuropsychologic symptoms such as deficits in memory, concentration, and spatial orientation are also observed in patients with Mn poisoning [1-3]. Unfortunately, the available treatment option for Mn poisoning is still very limited. L-3,4-dihydroxyphenylalanine (L-dopa) has been demonstrated possessing some therapeutic effects against Mn-poisoning but it fails to improve the parkinsonism-like symptoms such as walk time, tapping hand in manganese exposure welders [4]. Chelation agents like EDTA or para-aminosalicylic acid (PAS) have also been used to treat Mn poisoning. However, past studies have reported some conflicting results of EDTA about its therapeutic effects on manganism [5,6]. Therefore, it is very important to develop an effective therapy to prevent permanent neurological damages mediated by manganese at early stage.

Taurine is one of the free sulphur-containing amino acids that is widely distributed in human body, including blood plasma, heart, muscle, and brain tissue. Previous studies have reported that taurine has a neuroprotective effect against L-glutamate-induced excitotoxicity [7]. Taurine supplementation has been shown to benefit neuronal proliferation and synaptogenesis, suggesting its effects on enhancing synaptic plasticity and improving learning and memory [8]. Taurine has also been reported to improve the symptoms of metal-induced intoxication for lead and cadmium [9-11]. No severe side effects have been associated with taurine treatment. However, the effects of taurine on the learning and memory impairment induced by manganese exposure is still unknown.

In this study, we aimed to examine the role of taurine against Mn poisoning with a focus on the spatial learning and memory function in a rat model. We also explored the molecular mechanism of taurine in alleviating the Mn-induced neurological symptoms by evaluating acetylcholine synthesis and degradation. In addition, we conducted a systematic proteomic analysis by mass spectrometry to gain an insight into the genome-wide taurine-specific change in the protein expression profile. Our results provided novel evidences to support that taurine has a protective role in the Mn-induced neurotoxicity.

## Methods

### Animal study

60 male Sprague-Dawley rats weight (120 ± 10) g (approximately 6 weeks of age) were obtained from the experimental animal center of Guangxi Medical University (License No:SCXKGui2009-0002). All rats were supplied local tap water *ad libitum*, which contained the level of Mn that was below the detection limit. Rats were randomly assigned into 3 groups (20 rats/group): control, Mn, and taurine plus Mn group. Mn-administrated rats were injected intraperitoneally by 15 mg/kg MnCl_2_· 4H_2_O daily for 8 weeks. The rats in the taurine plus Mn group were injected intraperitoneally for 15 mg MnCl_2_· 4H_2_O/kg and 200 mg taurine/kg daily for 8 weeks. All rats were weighed daily to calculate the dosage of manganese and taurine treatment. Control animals received daily physiological saline injection for 8 weeks. All solutions were prepared with Milli-Q™ water. The procedures of animal experiments were in accordance with the protocols approved by the ethical committee for animal experiment of the Guangxi Medical University (GXMU-2014-036).

### Morris water maze

The Morris Water Maze was carried out within 24 h after last injection and was used to assess animal learning and memory ability [12]. The water maze used in our study was a flat black galvanized metal tank that was 210 cm in diameter and equipped with a platform 1–2 cm below the surface of the water. A camera was mounted above the maze and connected to a computer equipped with the Morris Water Maze analysis software (Huaibei Zhenghua biological equipment Co. China) to record the swimming track in the water maze. Before the learning trail, animals were placed on the fixed platform for 10s to familiarize themselves with the task. The animals facing to the pool wall were then trained to find the fixed platform from different locations (N, S, E, W) around the edge of the pool every day for 7 days. The trial was terminated once the animals reach the platform. If the animals failed to locate the platform within 90s, the animal was placed on the platform and the latency was recorded as 90s. The time(s), swimming distance (cm), and speed (cm/s) of escape latency to find the platform was used to evaluate the animal spatial memory. A surroundings and visual objects were positioned at fixed locations to serve as cues for the platform location. On day 8, a spatial probe trial was conducted with the original platform removed. The cumulative times spending in the original platform location was recorded during a period of 90s.

### Blood manganese level measurement

24 h after Morris Water Maze test, the animals were euthanized with pentobarbital (10 mg/kg). The blood of heart were immediately collected in polystyrene tubes which were previously washed by 10% HNO3 (Ultrapure grade, sigma, America) for 24 h and stored at -80°C until Mn analyses were conducted. 1 ml blood were digested with 3 ml HNO3 (65%) for 24 h at 130°C. The Inductively Coupled Plasma Atomic Emission Spectroscopy (ICP-AES) was used to analyze the level of manganese.

### Determination of acetylcholinesterase (AChE) and choline acetyltransferase (ChAT) activity

The measurement of AChE activity was based on the reaction of thiocholine, resulting from the enzymatic hydrolysis of acylthiocholines with 5, 5' dithiobis-2-nitrobenzoic acid (DTNB). The reaction solution was read at 412 nm and the enzyme activity was calculated in unit per gram of input protein. The activity of ChAT was determined by the ACh synthesis reaction that ChAT catalyzed (Acetyl-CoA + choline - > Acetylcholine + Co-enzyme A). The experiment were performed according to the instruction of the ChAT activity kit (Nanjing Jiancheng Bioengineering Institute, China). The wavelength of spectrometer reading was at 324 nm. The activity of ChAT was expressed in unit per wet weight of hippocampus.

### Protein sample preparation for rat hippocampus

Hippocampus of rats in the control, Mn and taurine plus Mn groups were dissected and lysed by the protein extraction buffer containing 20 mM Tris, 7 M urea, 2 M thiourea, 4% CHAPS, 10 mM 1,4-dithioerythritol, 0.5% ampholyte (4–7) and one tablet of protease inhibitors (Roche Diagnostics). Hippocampus were homogenized and centrifuged at 50 000 × *g* for 120 min. The supernatant were collected and the protein concentration in the supernatant was determined by the Bradford method (BioRad). 

### Two-dimensional gel electrophoresis

Isoelectric focusing was carried out by using the ETTAN IPGPhor apparatus (GE Healthcare) in Immobiline Dry Strip holders followed by approximately 750 μg protein loaded in ready strip. Briefly, a 24 cm pH 4-7 strip (GE Healthcare) was hydrated, focused, then equilibraten twice in equilibration buffer for 15 min. The IEF strips were placed on top of the 12.5% homogeneous polyacrylamide gels (26 cm-w 20 cm-h 1.0 mm-thick) in an Ettan DALT12 (GE Healthcare) gel apparatus. After the second-dimensional separation finish, the SDS-polyacrylamide gels were fixed and stained with silver nitrate. Gels were scanned on ProXpress CCD scanner (Perkin-Elmer). The images were then captured and analyzed by the Image Master 2D platinum 5.0 software, including background adjustment, landmark annotation, protein spot matching and points of difference analysis. At least three independent experiments were conducted for each group.

### Protein identification and analysis

The candidate protein spots were resected and dehydrated. The supernatant of rehydrated samples were then collected and analyzed in a time-of-flight mass spectrometer (Voyager DE-Pro Applied Biosystem). Peptide matching and protein searches were performed in the Swiss-prot database using MASCOT software. The analysis of the biological functions of identified proteins was based on the protein databases of Uniprot, Kegg pathway, Pathwe classification system, and literature search.

### Statistical analysis

Results were reported as mean ± SEM. The repeated-measures ANOVA was used for the analysis of the escape latency, swimming distance and speed among different groups. Bonferroni’s multiple comparison tests, one-way ANOVA followed by a correlation coefficient calculation were used for statistical analysis of probe times, blood manganese and AChE and ChAT activity. A *p* value less than 0.05 is considered as statistically significant.

## Results

### The effect of taurine in manganese-induced memory and learning impairment

**Figure 1:**
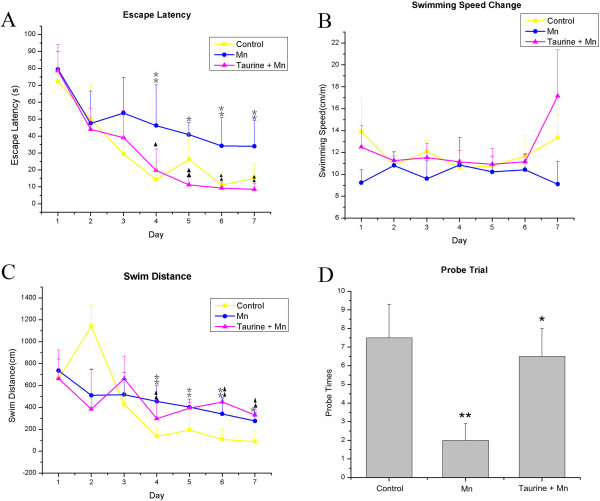
**Evaluation of rat learning and memory in the Morris Water Maze. (A)** Mean escape latency to the platform in the Morris water maze. **(B)** Mean swimming speed to the platform in the Morris water maze. **(C)** Mean cumulative distance in the Morris water maze. **(D)** Spatial memory retention in the Morris water maze probe trials among different groups. The test was conducted in the same way as the acquisition trials with the platform absent and 60s-trial limitation. The measure expressed is platform crossings ± S.E. Mn = 6-9. **p Mn versus control; *p < 0.05 versus Mn rats.

To test the effects of taurine in manganese-induced memory and spatial learning impairments, we performed the Morris Water Maze test. The acquisition trial of the MWM task is used to assess learning capacity, which is composed of the escape latency (time required to reach the platform) and the swimming distance to the platform. The probe trial is a method to measure the memory ability, which can be evaluated by the time spent in different areas of the pool or the number of target area crossings. A repeated-measure ANOVA revealed a significant difference in the escape latency (F(2,15) = 9.8, *p* < 0.05, Figure 1A) and swimming speed (F(2,15) = 16.8, p < 0.01, Figure 1B) but not the swimming distance (F(2,15) = 2.31, *p* > 0.05, Figure 1C) among the control, manganese and taurine plus manganese groups. The mean escape latency over a period of 7 days for the control group (31.3 ± 3.2 s) and taurine plus manganese group (30.1 ± 3.2 s) were significantly shorter than that of the manganese alone group (48.0 ± 3.2 s) (Adjustment multiple comparisons: Bonferroni, *p* < 0.01). The mean swimming speed of the control group (11.9 ± 0.3 mm/s) and taurine plus manganese group (12.2 ± 0.3 mm/s) was significantly faster than the manganese alone group (10.0 ± 0.3 mm/s) (multiple comparisons: Bonferroni, *p* < 0.05). In the probe trial on day 8, statistical analysis of the annulus crossings above the original platform position showed a significant improvement in the taurine plus manganese group compared to the manganese alone group (F(2,15) = 4.3, *p* < 0.05). Post hoc analysis indicated that the probe trial in the control and the taurine plus manganese group has an increase in the annulus crossings compared to that of the manganese alone group (control = 7.5 ± 1.8; taurine + manganese = 6.5 ± 1.5; manganese = 2.0 ± 0.9; least significant difference test, *p* < 0.05, Figure 1D).

### The effect of taurine on the blood level of manganese

**Figure 2:**
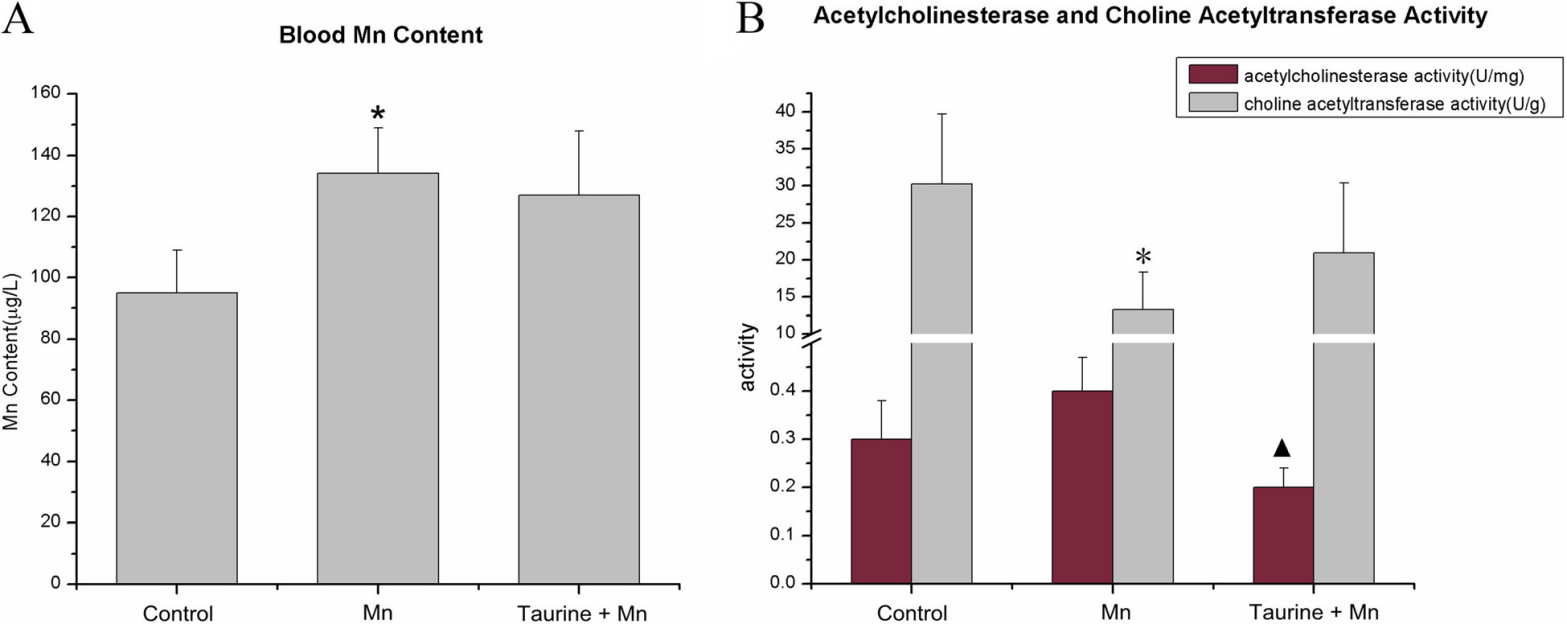
**Determination of manganese content in blood and the activity of acetylcholinesterase and choline acetyltransferase in hippocampus. (A)** Mean blood manganese level for different treatment groups (n = 8). * *p* < 0.05 versus control rats. **(B)** The comparison of acetylcholinesterase and choline acetyltransferase activity among different treatment groups (n = 8).

To test if the protective role of taurine in manganese-induced neurotoxicity is achieved by reducing the level of manganese in blood, we measured the blood manganese level by the Atomic Emission Spectroscopy. After 8 weeks of treatments, the blood manganese level was significantly higher in the manganese-administrated group than that in the control group (*p* = 0.000). However, the addition of taurine failed to reduce the blood manganese level, suggesting that taurine attenuates manganese-induced toxicity without affecting the level of manganese in blood (Figure 2A).

### The effect of manganese and taurine on the activity of acetylcholinesterase and choline acetyltransferase

The neurotransmitter acetylcholine has been long associated with the learning and memory in past studies. Because the level of acetylcholine is delicately regulated by the balance between the acetylcholinesterase (AChE)-mediated ACh degradation and the choline acetyltransferase (ChAT)-mediated ACh synthesis, we decided to measure the activity of both enzymes. The activity of AChE in manganese group (0.40 ± 0.26 U/mg) was not significantly different from that in the control group (0.29 ± 0.10 U/mg). However, the combined taurine and manganese treatment significantly reduced the activity of AChE (0.20 ± 0.04 IU/mg) compared to the manganese alone treatment (*p* = 0.032). The activity of ChAT was dramatically decreased after manganese treatment (control = 30.27 ± 9.47 IU/g; Mn = 13.24 ± 5.09 IU/g, *p* = 0.001). Administration of taurine (20.88 ± 9.50 IU/g) restored ChAT activity to 79% of control (Figure 2B).

### Proteomic analysis of the differences between the taurine and manganese treated rat hippocampus

**Figure 3:**
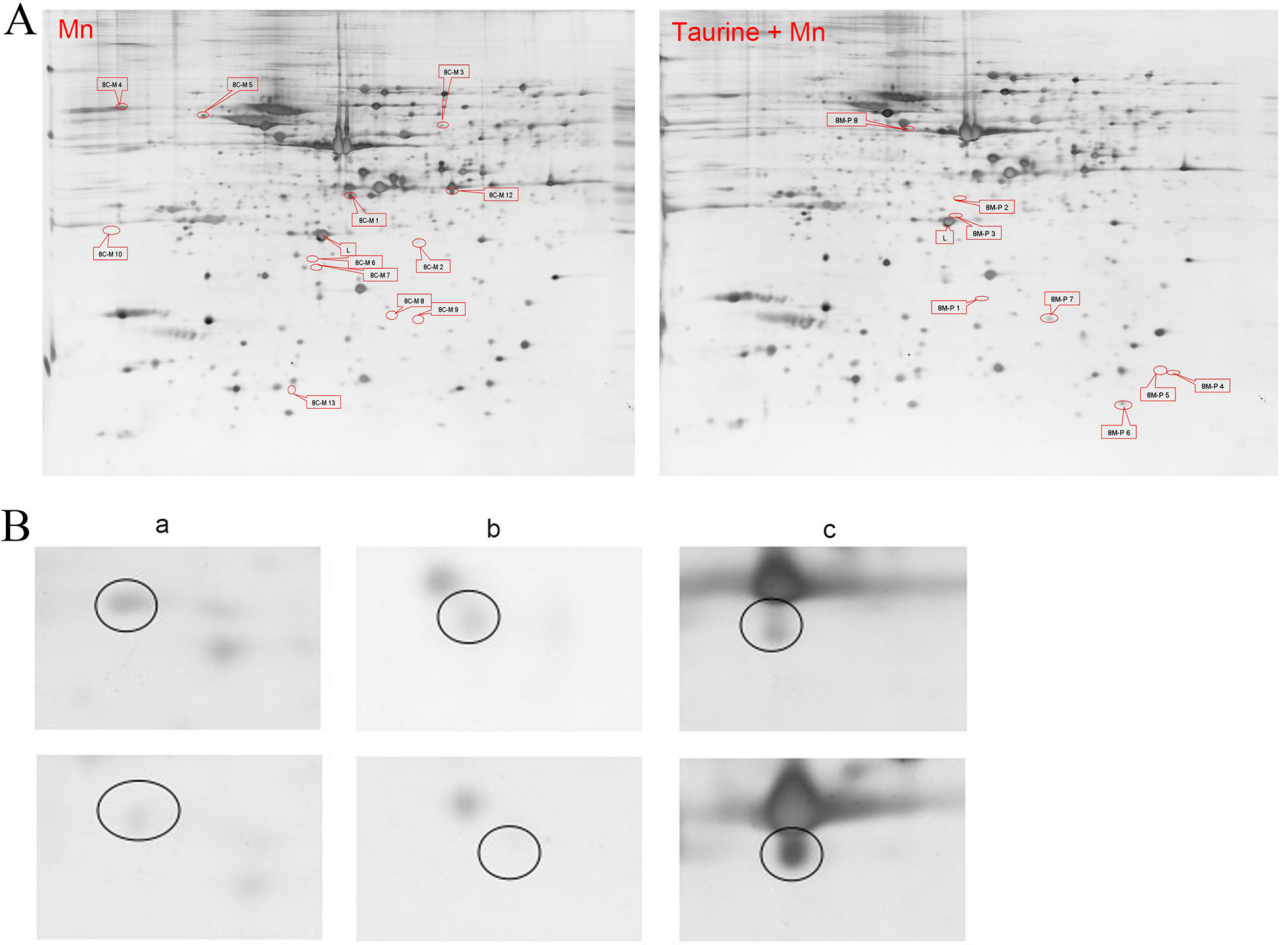
**Examples of differentially expressed proteins in hippocampus found by 2-DE. (A)** Representative 2D gel images from Mn and taurine + Mn. Molecular weight ranges from 250 KD to 10 KD and PIs ranges between 4 and 7. **(B)** Representative spots between the control and Mn treated groups. The control group as above line and 8w Mn administration group as below line. a(8C-M2):peroxiredoxin-6; b(8C-M8):dynactin subunit 3 isoform **B**; c(8C-M12):L-lactate dehydrogenase B chain.

We observed a total of 849 pairs of protein spots and 12 protein spots alteration when comparing the 2-D gels between the control and the manganese-treated samples (Figure 3A Mn). The identity of the 7 alterative spots were then revealed by mass spectrometry (L-lactate dehydrogenase B chain, mu-crystallin homolog, peroxiredoxin-6, actin-related protein 3 homolog, calreticulin precursor, secernin-1, dynactin subunit 3 isoform B). In addition, 785 pairs of protein spots were matched between the manganese-treated rat hippocampus and the manganese plus taurine treated rat hippocampus, while 2 alterative protein spots were successfully identified (prepro-A-type allatostatin(N) and glial fibrillary acidic protein delta, Figure 3A Taurine+Mn). The detail information of all proteins identified in this study was listed in Table 1.

**Table 1:** 9 identified viariation spots among different groups

**Spot no.**	**Protein identified**	**Accession no.**	**Mr(Da)**	**Protein PI**	**Protein score C.I.%**
8C-M 1	Mu-crystallin homolog	gi|16758840	33533.1	5.34	100
8C-M 2	Peroxiredoxin-6	gi|16758348	24803	5.64	98.70
8C-M 3	Actin-related protein 3 homolog	gi|161728791	47195.9	5.61	100
8C-M 4	Calreticulin precursor	gi|11693172	47965.8	4.33	100
8C-M 5	Secernin-1	gi|68163565	46366.6	4.73	100
8C-M 8	Dynactin subunit 3 isoform B	gi|227116275	17970.8	6.18	98.73
8C-M 12	L-lactate dehydrogenase B chain	gi|6981146	36589.1	5.71	100.00
8 M-P 4	Prepro-A-type allatostatin(N)	gi|152125945	15419.8	9.77	98.57
8 M-P 8	Glial fibrillary acidic protein delta	gi|5030428	48752.2	5.72	100

## Discussion

Taurine has also been shown to have neuroprotective effects in many pathological conditions such as Alzheimer’s and Huntington’s diseases [13,14]. However, the role of taurine in manganese-induced neurotoxicity has not been reported. Research has showed that accumulation of manganese in the brain disturbed the homeostasis of taurine by significantly increasing taurine uptake in rat astrocytes, suggesting a potential benefit of taurine in manganese overload [15]. In our study, Administration of 200 mg/kg/d of taurine significantly increased the ability of rats in the Morris Water Maze test. These results provided evidences to support the protective role of taurine in manganese-induced spatial learning impairment.

Previous research has shown that taurine formed less stable metal complexes with transition metals, such as Cu^2+^, Co^2+^, Cd^2+^, Fe^2+^, Mn^2+^, and Mg^2+^ than other amino acids [16]. In this study, we also examined whether taurine is able to chelate manganese from blood. Our result indicated that taurine had no effect on the blood manganese level, which is consistent to previous reports that taurine can’t remove lead from blood, kidney, liver and brain [17]. This results suggested that taurine reduces manganese-induced neurotoxicity without affecting the level of manganese in blood.

Taurine functions as a neuromodulator to attenuate glutamate-induced neurotoxicity in the central nervous system [18,19]. Numerous evidences have demonstrated that acetylcholine is strongly linked to the normal function of learning and memory [20,21]. The effects of manganese on the activity of AChE and ChAT, which are important indicators for the regulation of acetylcholine level in the brain, have been inconsistent [22-26]. We found that the manganese-induced increase in acetylcholine esterase activity and decrease in choline acetyltransferase activity were restored by taurine treatment, which is consistent with previous studies [27]. Muramatsu and his coworkers reported that taurine stabilized excitable membrane and suppressed the release of acetylcholine and norepinephrine at synapses [28]. In addition, the level of taurine is decreased in the brain of Alzheimer’s patients. Moreover, taurine supplement increased acetylcholine level in the experimental animal brain [29]. In a study by Hayate and his colleagues, taurine has been shown to restore the learning and memory deficits induced by intracerebroventricularly injection of streptozotocin by increasing the expression of ChAT in hippocampus [25]. All these evidences support that taurine improves the learning and memory function by controlling the level of acetylcholine.

In the proteomic analysis, we identified 9 differentially expressed proteins between the control, manganese, and taurine plus manganese groups. Among them, dynactin participates in the development of Huntington's disease, which is an autosomal dominant neurodegenerative disorder that affects primarily the striatum and cerebral cortex. The function of prepro-A-type allatostatin(N) is not clear yet. The rest of seven proteins all locate in cytoplasm and involve in the energy metabolism and oxidative stress pathways, implying that taurine serves as an antioxidant to protect the oxidative damages induced by manganese. Several lines of evidences have shown that taurine supplement protects oxidative damages induced by toxic compounds such as ethanol [25], lead [26] and β-amyloid [27]. Additionally, taurine is known to prevent oxidative stress by improving electron transport chain activity which is also responsible for ATP synthesis [28]. Mg competes with Mn to form MgATP, which serves as a substrate for ATPase in the synthesis of ATP [29]. Therefore, it is possible that taurine facilitates MgATP formation in the presence of Mn. Glial fibrillary acidic protein (GFAP) is a classic astrocyte marker and up-regulated upon exposure of many toxicants. Here, we observed that the expression of GFAP increased in taurine plus manganese group compared to the manganese alone group. However, some studies have indicated that taurine treatment was associated with a reduced level of GFAP in rats after 3-nitropropionic acid exposure in the xenotransplantated neurons derived from the human teratocarcinoma cell line [30]. This discrepancy could be resulted from the difference in the length of taurine treatment. Previous studies have shown that the expression of GFAP changes in a relatively slow rate [31,32]. Hence, it may take a longer treatment period before the expression of GFAP to decrease. The differentially expressed proteins we identified in this study still need to be further validated to elaborate their roles in the protective effects of taurine on cognitive deficit.

## Conclusions

In summary, taurine restores the activity of AChT and ChAT, which are critical for the regulation of acetylcholine. We have identified seven differentially expressed proteins specifically induced by manganese and two proteins induced by taurine from the rat hippocampus. Our results support that taurine improves the impaired learning and memory ability caused by excessive exposure of manganese.

## Competing interests

The authors declare that they have no competing interests.

## Authors' contributions

CLL, ST, ZJM and LYS carried out the experimental work. CLL, SCG and XYL designed the study, coordinated the experiments, analyzed data and wrote the manuscript. YYH and YPL conducted the statistical analyses. NM coordinated the experiments and manuscript modification. All authors read and approved the final manuscript.
